# Automatic Detection and Segmentation of Colorectal Cancer with Deep Residual Convolutional Neural Network

**DOI:** 10.1155/2022/3415603

**Published:** 2022-03-17

**Authors:** A. Akilandeswari, D. Sungeetha, Christeena Joseph, K. Thaiyalnayaki, K. Baskaran, R. Jothi Ramalingam, Hamad Al-Lohedan, Dhaifallah M. Al-dhayan, Muthusamy Karnan, Kibrom Meansbo Hadish

**Affiliations:** ^1^Department of Electronics and Communication Engineering, Saveetha School of Engineering, Saveetha Nagar, Thandalam, Chennai, India; ^2^Department of Electronics & Communication Engineering, SRM Institute of Science and Technology, Ramapuram, Chennai, India; ^3^Department of Electronics & Communication Engineering, Chennai Institute of Technology, Chennai, India; ^4^Surfactant Research Chair, Chemistry Department, College of Science, King Saud University, P.O. Box. 2455, Riyadh 11451, Saudi Arabia; ^5^Grassland and Forage Division, National Institute of Animal Science, Rural Development Administration, Cheonan 31000, Chungcheongnam-do, Republic of Korea; ^6^Faculty of Mechanical Engineering, AMIT Campus, Arba Minch University, Arba Minch, Ethiopia

## Abstract

Early and automatic detection of colorectal tumors is essential for cancer analysis, and the same is implemented using computer-aided diagnosis (CAD). A computerized tomography (CT) image of the colon is being used to identify colorectal carcinoma. Digital imaging and communication in medicine (DICOM) is a standard medical imaging format to process and analyze images digitally. Accurate detection of tumor cells in the complex digestive tract is necessary for optimal treatment. The proposed work is divided into two phases. The first phase involves the segmentation, and the second phase is the extraction of the colon lesions with the observed segmentation parameters. A deep convolutional neural network (DCNN) based residual network approach for the colon and polyps' segmentation from the CT images is applied over the 2D CT images. The residual stack block is being added to the hidden layers with short skip nuance, which helps to retain spatial information. ResNet-enabled CNN is employed in the current work to achieve complete boundary segmentation of the colon cancer region. The results obtained through segmentation serve as features for further extraction and classification of benign as well as malignant colon cancer. Performance evaluation metrics indicate that the proposed network model has effectively segmented and classified colorectal tumors with dice scores of 91.57% (on average), sensitivity = 98.28, specificity = 98.68, and accuracy = 98.82.

## 1. Introduction

Colon/colorectal cancer is the second leading cause of cancer-related deaths in men and the most commonly detected cancer worldwide. As per the statistics of the American Cancer Society, survivors of colorectal cancer are 65.4%, less than when compared to breast cancer (90.35%) and prostate cancer (99.6%) [1]. The polyps covering the lower end of the digestive tract are the reason for colon cancer. Detection of polyps at an early stage may increase the survival rate of the diseased from 10–15% to 60–80% [2]. Two-dimensional imaging technologies such as computed tomography (CT) have been involved in the detection of colorectal cancer. These CT scan images, obtained by radiology techniques, reduce the death rate of colon cancer by 20%. MRI results have been proven to produce better accuracy compared to CT-scanned images, but the cost factor of CT scan is almost four times less than MRI. This has made CT scan images serve better for developing countries [3, 4]. The developments in the scanning technology of CT have resulted in a large number of electronic health records, which has become a substantial challenge for the diagnosis of colon cancer by the radiologist. In addition, the detection and diagnosis of colons or polyps from large data are highly time-consuming and hectic for human intervention. Hence, to increase the detection speed and to ease the burden, the radiologists' computer-aided diagnosis (CAD) system is involved [5].

Colon segmentation is the initial stage of the CAD processed image. Many algorithms have been evolving to segment colon cancer from CT images [6, 7]. Better segmentation is still the need of the hour of locating the polyps accurately. Researchers have identified this technology of region growing helps to identify the size, shape, and texture of the polyps contained in the intestine [8]. The methods of the energy optimizer, inclusive of level set [9], graph-cut [10], were also observed. Despite all these technology developments, the accuracy and robustness of identifying colon cancer continue to be a big challenge, particularly when polyps resemble most likely to the intestinal region. However, for morphological operation, the structuring element dimensions vary depending on the size and shape of the polyps' development. The segmentation techniques proposed to extract colon and polyps from abdominal CT images are broadly classified into four categories: morphological operation, region growing, optimization, and machine learning methods. Cancer segmentation enabled through morphological methods has evolved in the past as the morphological operation involved the removal of vessels attached to polyps along with the connected component. With fixed size, morphological operator segmentation becomes difficult [11] as the polyps' vessel radius reduces when the vessels evolve along the periphery of the liver.

However, the proposed methods were simple for implementation; the different sizes of colon lesions make it difficult to fix the size of the morphological operator. The uneven size template of the morphological operator leads to poor segmentation of large-sized lesions and overfitting of small-sized polyps. In addition, nonsolid polyps are challenging to morphological templates [12]. According to nonsolid colons which are challenging for morphological operations, each pixel in the image is adjusted based on the value of other pixels in its neighborhood. A user initializes a seed point in the region growing method to get colon segmentation. To satisfy the convergence measure of adding internal voxels into bowel in region growing method, the surrounding regions and polyps or colons appear similar, which can be isolated using the above method. Contrast-region growing methods have been utilized in colon segmentation [14]. Probability determination of every voxel belonging to colon tumors is done for the intensity values after which the region growing is applied to separate background regions and the colon. The convergence measure in the region growing technique is highly challenging due to the asymmetrical shape of the colon. Later, energy optimization techniques such as level and graph-cut methods were involved in colon cancer segmentation. The methods of Chan–Vese and level set were used for image description, and the corresponding function sinks its value when the abdominal boundary and contour segmentation match. The level set technique was used for dimensioning the shape before the hypothesis [9]. Colon segmentation by the graph-cut method reframes the design problem into an optimization task. This energy optimizer performs better for isolated colons but not in the case of polyps and ground glass polyps. A set of highly informative features can be extracted using advanced machine learning techniques. With these features set, high-level classifiers can be used for classification. The feature set beholds the shape and texture voxels of 2D images, which can be classified using conditional random-field classifiers. Nowadays, the most common machine learning technique, called deep learning models, has been involved in medical image classification.

**Figure 1:**
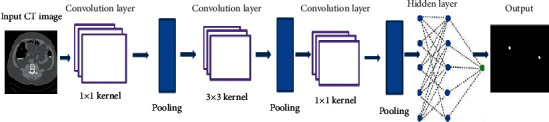
Deep convolutional neural network architecture.

This deep learning network provides good classification by learning various features in the image [14]. The self-learning capability and hidden layer combination of DCNN have produced good results in medical image processing. Previous medical image segmentation of neuron membranes has been shown to produce better results using end-to-end segmentation [15]. Many approaches, along with convolutional networks, have evolved to produce better classification results, one of which is UNET [16], where the detection object is done to produce morphological details of the identified object. Combining a deep learning network with a level set for lung nodule segmentation is highly challenging [17]. Hence, many researchers have arrived at various colon cancer segmentation techniques until now, some of them are not fully automated and few require expert intervention. These algorithms [18] do not solve the problem of identifying polyps by separating them from their surrounding intestinal regions. By using the level set technique, autosegmentation of the colon is highly challenging [19]. In the current work, a deep convolutional neural network architecture is proposed with connections like long as well as short skips for automatically segmenting colon cancer. The DCNN network architecture has adopted an end-to-end classification scheme [20] for efficient diagnosis of colorectal tumor. Enhanced performance of the currently proposed network architecture is attained by making deep layer connections across the pooling blocks. The input information is exchanged within the convolutional layers of the deep layer connection along with the combination of long and short connections in the output. The training phase accuracy and the classification efficiency of the network are found to enhance the smooth flow of information in the proposed architecture. In the current work, two different types of tumor regions are diagnosed, namely, solitary polyps and colons. The present work aims to identify two different types of tumor regions that are diagnosed in the current work, namely, solitary polyps and colons amidst the surrounding tissues. The schematic approach in Figure 1 of the proposed architecture helps to solve the difficulty of detecting the polyps from its background region. Precise segmentation of colons is attained on 2D CT images using the DCNN network in the current work. Training time elapsed for 2D images is comparatively less than that of 3D image data. The algorithm followed in the present work can be summarized as follows:

- The deep convolutional neural network proposed in the current work achieves better accuracy in the segmentation of colorectal cancer, especially in colons or polyps, with no necessary postprocessing.
- The spatial information and complete resolution features are preserved in the proposed convolutional network, which has been modelled in such a way to increase the convergence speed within the deep networks.

## 2. Materials and Methods

### 2.1. Datasets

**Figure 2:**
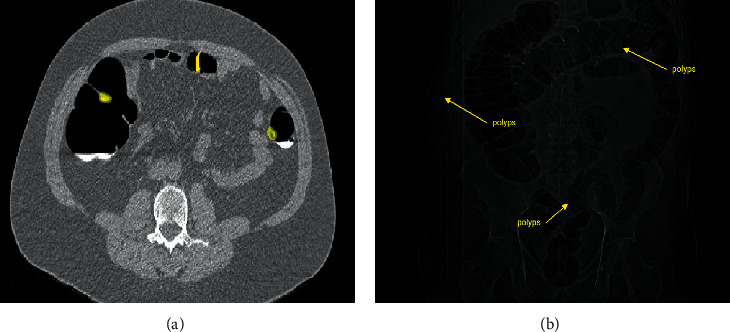
(a) Sample input slice. (b) Its corresponding bowel cavity.

Input data have been accessed from a public dataset called The Cancer Imaging Archive (TCIA) public access/CT Colonography [21]. The TCIA database contains 825 cases of patients, and 941771 CT scan images are available with details of .xls and the format of DICOM images. The CT images in this database provide details such as polyp description and location of the colon segments as shown in Figure 2. Computed tomography (CT) colonography or virtual colonoscopy uses special x-ray equipment to examine the different size and shape of the large intestine for cancer and growths called polyps. The colon and polyp sizes were annotated by different radiologists as <9 mm and lesions >10 mm. The subsequent combination of blinded and unblinded reviews, the XMS format of 893 colonial lesions (ground truth), is designated and available for use. The TCIA paper [22, 23] can be referred for more particulars on this. In the present work, the overall dataset has been randomly divided into three subsets: (training set) 1243, (validation set) 189, and 86 (testing set).

### 2.2. Network Architecture

**Figure 3:**
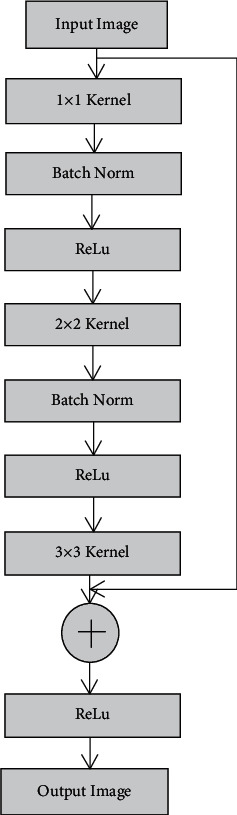
Residual network architecture.

The overall flow of the network model involved in the present work is given in Figure 1. The convolutional residual neural network [24] rightly triggers convolutional encoding and decoding actions. Choosing a small pattern (kernel) and recognizing its occurrence from the input image is carried out through convolution. The same pattern/features of the input colorectal CT image are fed into the transform and can be retrieved back through a convolutional network structure [25]. This part defines the boundary for the desired region of interest to extract the features by segmentation. Multiple resolution levels (multipixel) are retained within the image features, which is made possible through multiscaling made available through convolutional and residual blocks. The network model is built with numerous blocks, and the specific arrangement of the residual blocks is depicted as in Figure 3. Filters of the triconvolutional layer are arranged as 1 × 1, 3 × 3, and 1 × 1 fashion, respectively. An activating unit called ReLu (rectified linear unit) follows each of these convolutional layers. In between the convolution and ReLu layers lies the batch norm layer, whose purpose is to minimize the internal covariance shift and to speed up the training process.

The max pooling layer contained within the convolution layer is responsible for performing sampling of the colorectal CT image. The CT colon images of different pixels are organized by pooling, where the images are labelled based on their pixel probability. The colon region is carefully segmented from the abdominal region using ResNet 50 in the current residual network model. Loss of spatial detail has to be conserved after the pooling effect, which is achieved by the residual and convolutional blocks fitted with short and long skips, respectively. The residual block fitted with a short skip is directly connected to the previous layer without any interruption. This type of nested arrangement (long and short skip connectivity) helps in the hassle-free flow of information from one layer to the other. The advantage of the residual-based convolutional model is that it accepts raw input images (as available in the dataset) to implement segmentation to extract its features.

### 2.3. Deep Convolutional Neural Network

DCNN, in combination with transfer learning [26], has been involved in the present architectural model for colon cancer segmentation. Stacking number of deep network model visions better classification under challenging environments but appears difficult in the training of these stacked layers. Hence, the training of shallow network models is much easier than that of deep network layers. Keep on adding more layers in the network can also cause the network convergence to degrade. This is where the hidden layers appear, and the degradation problem has been seen to be overruled. Incorporation of more hidden layers has given promising results with better efficiency and overcomes degradation problems compared to other network models such as ImageNet and MSCOCO [27]. A deep residual layer with a skip connection in this architecture enables better optimization [28]. This residual layer is used to build the network model and is shown in Figure 2. The hidden layer in the deep residual network plays a vital role in the transmission of information between the blocks with the help of skip connectivity. Filters of the triconvolutional layer are arranged as 1 × 1, 3 × 3, and 1 × 1, and the batch norm layer follows each of the convolution layers. The residual layer network follows the learning function as given in the following equation:

(1) $$\begin{array}{c} x_{l}+1=x_{l}+r\left(x_{l},w_{l}\right). \end{array}$$

Here, *r* stands for the residual function (batch norm layer stacked between two convolutional layers).

*x*_*l*_ is the input feature vector, and *x*_*l*_+1 is the output feature vector of the *l-*th residual network.

*w*_*l*_ is the total number of weights and biases of the *l-*th residual network.

## 3. Results

**Figure 4:**
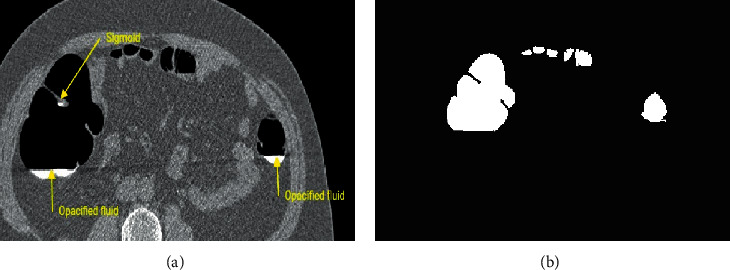
(a) Original CT image (axial slice). (b) Segmentation of colon air pockets.

Current work of the deep residual convolutional neural network was evaluated on the TCIA database images. The images were acquired using 12 datasets from TCIA, with each of the 6 images being in supine and prone positions. Colorectal cancer is detected with the help of this proposed segmentation technique through polyp identification found over the walls of the colon. Thus, the opacified fluid collection and sigmoids identified over the intestinal wall and its corresponding air pocket segmentation are shown in Figure 4. The proposed method results in automatic segmentation for the removal of bowel and segmentation of the colon into its surrounding regions are identified.

**Figure 5:**
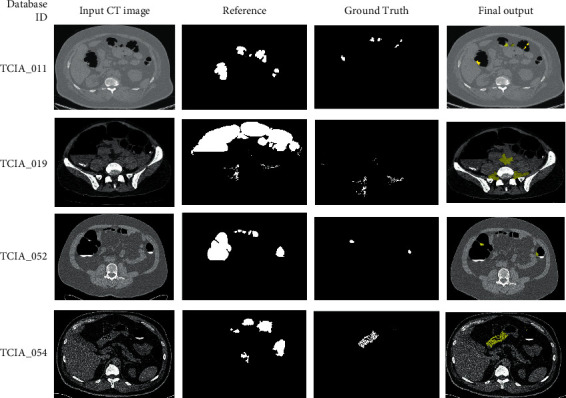
Segmentation results.

The inputs are downsampled nonlinearly using pooling blocks (this is established by subjecting them to the max function). The effect of overfitting is reduced with the help of the double effect, which reduces the number of parameters in the network and results in representing the image being spatially invariant. The representation is made possible through network learning by the local pattern descriptors. The depth of the network is decided by the number of convolution and pooling blocks that can be related to the hierarchical representation of the image. The training model for the proposed DCNN network involves an iterative process that takes the entire dataset through many passes until the model converges. At each step of the training phase, data flows from layer by layer from the first to last to find the classification error and loss function. There is a possibility of this error flowing backward in the network, and tuning of the network weights minimizes the training data's classification error. The segmentation using the proposed architecture is given for some sample images in Figure 5.

### 3.1. Evaluation Parameters

The quantitative measure of the current technique has been evaluated using a metric called the dice coefficient (DC). This metric evaluates the parameters based upon the variance calculated between the automatic segmentation features and manually annotated (used as reference) segmentation results. The following equation defines the computed DC as the relation between the intersecting binary masks and the actual number of elements present in every set.

(2) $$\begin{array}{c} \text{DC}=2\frac{\left(A_{s}\cap B_{m}\right)}{\left|A_{s}+B_{m}\right|}. \end{array}$$

*A*_*s*_ is assumed to be binary-mask in the proposed technique.

*B*_*m*_ is a binary-mask of the manually annotated segmentation results.

The intersection results of DC occupy the value between [10, 1] practically, whereas in theory, the value of 1 refers to fully overlapped and the value of 0 refers to zero overlap between the segmented output and the reference [29, 30]. Sensitivity is defined by the following equation:

(3) $$\begin{array}{c} \text{sensitivity}=\frac{\text{TP}}{\text{FN}+\text{TP}}. \end{array}$$

The equation for specificity can be defined as in the following equation:

(4) $$\begin{array}{c} \text{specificity}=\frac{\text{TN}}{\text{FP}+\text{TN}}. \end{array}$$

The equation of accuracy is defined as given by the following equation:

(5) $$\begin{array}{c} \text{accuracy}=\frac{\text{TP}+\text{TN}}{\text{FP}+\text{FN}+\text{TP}+\text{TN}}, \end{array}$$

where TP (true positive) is the actual number of colon regions rightly detected, TN (true negative) is the number of pseudo-colon regions identified as actual colons, FP (false positive) is the missed number of actual colons, and FN (false negative) is actual number of pseudo-colon regions rightly detected.

**Figure 6:**
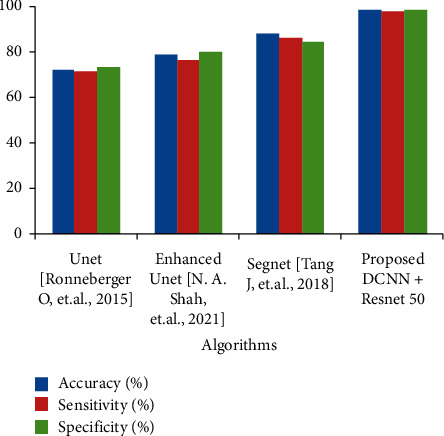
Performance comparison between conventional and proposed techniques.

The detection module is trained with a mixture of multiresolution CT images of 243 and test images of 86. The percentage of accuracy achieved with the currently proposed technique is validated as a training versus test dataset as shown in Figure 6. The proposed DCNN with residual network architecture has been trained using the stochastic gradient descent technique. The 2D CT input images are set to 512 × 512 resolution size to serve the purpose of testing and later prediction. The rate of learning is fixed up to 0.0001 with the available models and trained for 50,000 iterations, which results in an overfitting problem. The weights for the decay are fixed at 0.0005 with a momentum value of 0.8 and a total of 7 hours to train the entire network.

## 4. Discussion

Current work involves the input data from the TCIA dataset [21] that includes different colon varieties with diverse sizes, shapes, and textures, present at various locations in the liver metastasis. Comparison between the state of art techniques over the colon cancer segmentation obtained with TCIA dataset images that contain a tumor in brain MRI images while there are other images in the Kaggle dataset. The conventional algorithms in the literature, namely, level set, graph-cut, and UNET [31], were observed to include the dice coefficient as a key parameter indicator for convolutional and deconvolutional deep networks [32]. Here, in the current work, the performance analysis was measured through the DC parameter.

**Figure 7:**
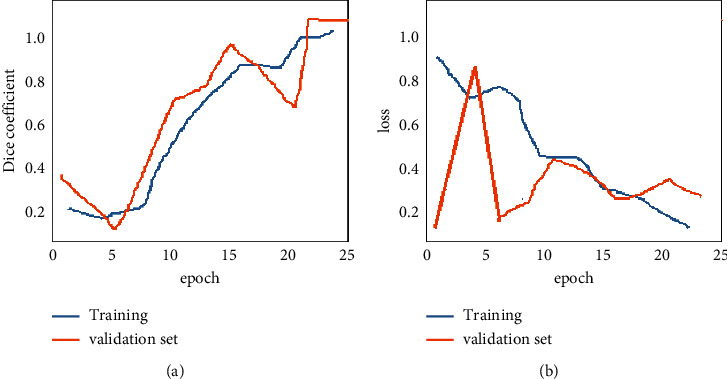
(a) Dice coefficient (DC) curve and (b) cross entropy loss curve for training and validation set data.

Our method has achieved a DC of 91.57% over the TCIA database, which is higher than the other state-of-the-art methods as given in Figure 7; the deep network model also effectively segments the colon cases, including polyps in colonial regions. The segmented polyps or colons from the bowel regions are shown in Figure 5. The plot depicted in Figure 6 shows the qualitative performance of various techniques proposed already in the past for colon cancer segmentation. The comparison shows that the current work using ResNet has achieved better performance than the others have. This is because conventional techniques do not differentiate the polyps from the background bowel regions. In addition, the problems of underfitting and overfitting of the morphological operators can be avoided with the proposed technique. The accuracy of the present work's results motivates the development of the same for 3D input CT images [33]. The results include better accuracy, sensitivity, and specificity for the current work compared to its previous techniques.

The unsupervised colon cancer segmentation method [34] required less manual intervention. However, the level of accuracy obtained is typically less than that of the present technique. Another method of invasive endoscopic technique provides information on the digestive track [35], but it appears most uncomfortable for the patients; hence, we chose CT scan images in the present work. In addition, the endoscopic technique needs to be performed under the surveillance of medical expert personnel and thereafter followed by tumor segmentation. The proposed residual-based learning network also overcomes the degradation error during the training of deep neural networks. The ResNet in the present work is built on 50 deep layers to identify the representative features for colon cancer region segmentation. The results produced by the present work state that the accuracy improves with no need for any postprocessing.

The modern technique of deep learning has been trying to bring out reliable features to differentiate the effects due to partial volume present in fecal material and thin soft tissue layers lying in between the two tagged regions. The difficulty is that the soft-tissue layer is distorted due to pseudo-enhanced artifacts and results in soft-tissue layers, which produce partial volume effects of fecal material [36]. The training sample numbers included in these techniques are very small; hence, comprehensive sample numbers are required to overcome such artifacts.

Automatic detection of colon tumors was studied earlier with artificial intelligence-based neural network models. The model was a single-shot detector to identify the lesions from colonography videos recorded from 24 patients with an accuracy rate of 93.5% (on an average between two sets of training and testing) [37]. The present model has achieved better accuracy and validation momentum with a minimum number of epochs. Also, a similar previous study based on supervised machine learning technique was involved for classification of various types of polyps. The study focused on automatic segmentation of individual polyps has attained an accuracy value of 90.18% and 83.37% (on an average) with the augmentation of two datasets [38]. These results are far behind the level of accuracy we have achieved in polyp detection and classification, which is 98.82%.

## 5. Conclusion

Automatic detection of colon cancer is implemented in the present work through segmentation and classification of the abdominal region. The beneficial digital image processing technique has helped to identify the colonic region in abdominal 2D CT images. The deep residual convolutional neural network has efficiently segmented various-sized polyps from the challenging colonic regions. Colorectal cancer images are detected correctly through proper segmentation and have overcome the inherent obstacles due to uneven stacking of residual layers. The input image features are learned by the proposed end-to-end deep network technique automatically. The relative information of the feature set flow between the convolutional layers smoothly eased the strong detection of colon/polyps from its background. The detection and classification of cancer-induced polyps are qualitatively analyzed with evaluation metrics such as accuracy, specificity, and DC. The current work has enhanced the probability of identifying colon cancer and can serve as a better reading tool for clinical data, which can reduce the manual reading time to serve the clinicians better.

## Data Availability

The data used to support the findings of this study are included in the article. Should further data or information be required, these are available from the corresponding author upon request.
